# INCRESE: Development of an Inventory to Characterize Recorded Mental Health Recovery Narratives

**Published:** 2020

**Authors:** Joy Llewellyn-Beardsley, Skye Barbic, Stefan Rennick-Egglestone, Fiona Ng, James Roe, Ada Hui, Donna Franklin, Emilia Deakin, Laurie Hare-Duke, Mike Slade

**Affiliations:** 1 School of Health Sciences, Institute of Mental Health, University of Nottingham, Nottingham, United Kingdom; 2 Department of Occupational Science and Occupational Therapy, Faculty of Medicine, University of British Columbia, Vancouver, Canada; 3 National Institute for Health Research, ARC East Midlands, University of Nottingham, Nottingham, United Kingdom; 4 NEON Lived Experience Advisory Panel, Nottingham, United Kingdom

**Keywords:** Mental Health, Recovery Narratives, Content Analysis, Diversity, Inventory, Reliability

## Abstract

**Objective:**

Mental health recovery narratives are increasingly used in clinical practice, public health campaigns, and as directly-accessed online resources. No instrument exists to describe characteristics of individual recovery narratives. The aims were to develop and evaluate an inventory to characterize recorded recovery narratives.

**Research Design and Methods:**

A preliminary version of the Inventory of Characteristics of Recovery Stories (INCRESE) was generated from an existing theory-base. Feasibility and acceptability were evaluated by two coders each rating 30 purposively-selected narratives. A refined version was produced and a formal evaluation conducted. Reliability was assessed by four coders each rating 95 purposively-selected narratives. Inter-coder reliability was assessed using Fleiss’s kappa coefficients; test-retest reliability was assessed using intra-class correlation coefficients (ICCs).

**Results:**

Multiple refinements to description, coding categories, and language were made. Data completeness was high, and no floor or ceiling effects were found. Intercoder reliability ranged from moderate (k=0.58) to perfect (k=1.00) agreement. Test-retest reliability ranged from moderate (ICC=0.57) to complete (ICC=1.00) agreement. The final INCRESE comprises 77 items spanning five sections: Narrative Eligibility; Narrative Mode; Narrator Characteristics; Narrative Characteristics; Narrative Content.

**Conclusion:**

INCRESE is the first evaluated tool to characterize mental health recovery narratives. It addresses current concerns around normative recovery narratives being used to promote compulsory wellness, e.g. by identifying narratives that reject diagnosis as an explanatory model and those with non-upward trajectories. INCRESE can be used to establish the diversity of a narrative collection and will be used in the NEON trials (ISRCTN11152837, ISRCTN63197153, ISRCTN76355273) to allow a recommender system to match narratives to participants.

## Introduction

The mental health recovery narratives of individuals (hereafter ‘recovery narratives’)^1^ are a central component of clinical practice and other recovery-oriented interventions, for example narrative-based therapies,^2–4^ Recovery College courses,^5,6^ and peer support.^7,8^

Recovery narratives that have been recorded by others are increasingly used to augment this individual support. Recorded recovery narratives have been defined as first-person non-fiction accounts of recovery from mental health issues, including elements of adversity or struggle, and of self-defined strengths, successes, or survival.^9^ They are widely available in the media, for example, memoirs and documentaries, and are used within mental health services in various forms, such as written and audio materials in bibliotherapy,^10,11^ lived experience videos in digital interventions,^12^ and blogs on national mental health organization websites. A publicly-available example fitting this definition is “Making Recovery Real in Dundee: Rona’s Story,” available at https://youtu.be/7kGMazsGDJw. Recovery narratives are also used in wider health contexts such as public health and anti-stigma campaigns.^13,14^ Yet, until recently there has been little research on the impact of recovery narratives on those who access them (hereafter narrative ‘recipients’).

Evidence about impact on recipients is now emerging. A systematic review of five studies showed that recovery narratives can be both beneficial and harmful to recipients experiencing mental health issues.^15^ A large-scale interview study found that positive impacts on recipients included feeling increased connection with others, validation of own experiences; empowerment and hopefulness about the future; greater life appreciation; changes in perspective; and a reduced sense of stigma. Negative impacts included a sense of inadequacy; disconnection from others; increased pessimism; and feeling emotionally burdened by others’ distress.^16^ Factors mediating whether a narrative had a beneficial or harmful impact on a recipient included recognizing shared experiences or personal similarities with the narrator, which led to positive outcomes, and noticing narrator achievements, which led to hope and empowerment for some but inadequacy and disconnection for others.^16,17^

Clearly, not all narratives help all people at all times. How then can practitioners, public health campaigners, or individuals assess which narratives may be most beneficial, while avoiding potential harms? Although research has been undertaken in related areas, for example, whether content warnings are helpful in avoiding harms for some recipients,^18,19^ no existing guidance describes what kinds of recovery narratives might have positive impacts for individuals, and how potential harms may be avoided.

Evidence is emerging, however, of the narrative characteristics that may affect a recipient’s effective connection to a narrative, such as having shared experiences or the perceived authenticity of the narrator.^20^ This evidence replicates findings in other fields. For example, reception theory, originating in communication and media studies, suggests that recipients are not passive but bring their own readings to narratives. A narrator may encode their narrative with various meanings, but the receiver may decode and interpret the narrative in different ways, relevant to their own circumstances such as individual cultural background and life experience.^21^ Relatedly, narrative transportation theory suggests that recipients are more fully absorbed by narrative worlds if they can identify with aspects of the narrative or narrator.^22,23^ And the more fully absorbed or transported they are, the more likely they are to be persuaded by a narrative’s messages^24^ – in this case, that recovery of a meaningful life may be possible for them.

Since characteristics of a narrative influence whether a recipient is likely to connect effectively with its message, an approach to identifying and describing the characteristics of a recovery narrative is needed. This will enable individuals to differentiate between narratives in advance of accessing them fully, and to choose narratives with characteristics that may be most helpful at a particular time.

No instrument currently exists to characterize recovery narratives. Available narrative-related instruments within mental health are designed to assess a narrator’s mental processes or emotional states through their narratives, such as a sense of self in the Scale To Assess Narrative Development (STAND)^25^ or narrator coherence in the Narrative Coherence Rating Scale.^26^ Characterizing a narrative itself requires a different tool, drawing on methods used in other fields, such as the analysis of media content. An example is the PICMIN instrument (Picture of Mental Illness in Newspapers), which assesses mental illness stigma in print media.^27^ PICMIN was developed using principles of content analysis, a systematic, replicable technique for compressing large amounts of text into fewer content categories based on explicit rules of coding, for use with qualitative and quantitative data.^28^ Content analysis has been extended to health research, for example, in investigating stigmatizing attitudes towards mental health in social media^29^ and exploring coping strategies for withdrawing from anti-psychotic medication.^30^ It provides a set of procedures for the systematic coding of data, described by Robson^31^ as a six-stage approach: identifying the research question; selecting the sample; defining the unit of analysis; constructing the categories for analysis; testing the categories on samples and assessing reliability; and conducting analysis. In this study, we adopted these procedures in modified form as design principles, omitting the final stage.

Content analysis was originally used to identify manifest (visible, surface-level) content, but has widened to include the identification of latent content, concerning underlying patterns or deep-level inferences.^32,33^ Connection with a narrative is a complex process, involving recipient judgements about multifaceted phenomena such as authenticity.^20^ Therefore, the characterization of latent as well as manifest content is needed, to ensure sufficient depth of characterization is achieved.

Any characterization of narratives risks adopting a reductive approach to its source material, and clearly a list of characteristics cannot replace the complexity of a narrative. What is needed is a ‘condensation’ - a process of shortening an artefact while still preserving its core^32^ - to provide an instrument that may assist individuals and practitioners in finding their way to stories that might be most helpful for them.

Critical theorists and survivor/user researchers have also raised concerns about the commodification of recovery narratives within mental health practice,^34^ for example when only certain “highly circumscribed” or “normatively successful” kinds of stories are promoted by services,^35^ or when stories are used to promote “compulsory wellness.”^36^ Since evidence suggests that people connect with narratives they can identify with,^22–24^ and since connection is the key mechanism of impact,^16^ a tool that can characterize maximally diverse types of recovery narrative is required to create benefits for the greatest number of people. This study is therefore based on theory that uses the widest possible definitions of recovery narratives and which has been tested on diverse populations including marginalized groups and those currently under-served by mental health services.

The aim of this mixed methods study was to develop and evaluate an inclusive and in-depth inventory to characterize recorded recovery narratives, for use by mental health practitioners, individuals experiencing mental health distress, researchers, and curators of narrative-based collections or interventions. The objectives were to assess the inventory for its statistical reliability, and to assess its feasibility and acceptability to two groups: (1) a team of coders, as representatives of inventory target users; (2) a panel of people with lived experience of mental health difficulties, as representatives of the group whose stories would be characterized by the inventory.

## Research Design and Methods

### Ethical Considerations

The research was undertaken as part of the Narrative Experiences Online (NEON) study. NEON aims to examine whether engagement with the recovery narratives of others can influence an individual’s own recovery journey. Findings will inform three future trials (ISRCTN11152837, ISRCTN63197153, ISRCTN76355273).

Ethical Committee approval was obtained in advance for a protocol describing procedures for working with narratives and coders of narratives (London-West London REC and GTAC 18/LO/0991). The protocol for narratives described procedures for collecting consent for usage of narratives. The protocol for coders applied to all contributors not employed as NEON researchers. It described procedures for informed consent and to address the emotional and physical needs of research participants. All coders classified as research participants were provided with information sheets, and met with a member of the research team prior to study commencement to provide written informed consent. Procedures for emotional and physical wellbeing took account of emotional labour, given the potentially distressing content of some recovery narratives, and the possibility of fatigue through coding large numbers of narratives.

### Stage 1: Development of Preliminary INCRESE

A theory-based list of candidate inventory items and responses was generated through inspection of: a conceptual framework for recovery narratives;^1,9^ change models of helpful and harmful recovery narrative impact^15,16^ and recipients’ experiences of hope and connection;^20^ risk factors for developing mental health issues;^37^ research on turning points in recovery narratives;^38–44^ research on recovery factors;^45^ and research on content warnings.^18, 46–50^ Demographic item responses incorporated guidance from the UK Office of National Statistics for narrator ethnicity,^51^ Stonewall UK for narrator sexuality,^52^ NHS England for disability,^53^ and the Diagnostic and Statistical Manual of Mental Disorders for items characterizing diagnosis.^54^ This produced the preliminary inventory, INCRESE v1.

### Stage 2: Pilot Evaluation

The aim of the pilot evaluation was to test the feasibility and acceptability of INCRESE v1 to coders. Feasibility was defined, modifying an existing definition, as ‘suitable for use on a routine, sustainable and meaningful basis by target users when used in a specified manner for a specified purpose.’^55^ The acceptability of a tool to its target users is important for successful implementation.^56^ Use of appropriate language and a reasonable level of administrative burden have been identified as important factors in achieving this.^55^ Acceptability was thus defined as ‘capturing sufficient characteristics to provide an accurate summary of a recovery narrative, while representing reasonable administrative burden and using appropriate language.’ Reasonable administrative burden was defined as each narrative taking <15 minutes to code. Appropriate language was defined as sensitive, inclusive, and acceptable both to coders and people with lived experience of mental health distress.

INCRESE v1 was tested by research team coders (JLB and FN) from sociology and psychology backgrounds, each of whom independently coded 30 narratives twice, two weeks apart. The narratives were a purposive sample,^57^ selected for maximum variation in format (e.g., text, video), form (e.g., prose, poetry) and length. Characteristics of recovery narratives may differ according to form,^1^ so text-based, audio and video narratives were chosen to test whether INCRESE could be applied equally to narratives across formats and forms. Narratives varied in length to test whether INCRESE sufficiently captured characteristics of both longer and shorter narratives, and whether administrative burden for longer narratives remained acceptable. Very long narratives such as documentaries and memoirs were excluded, due to limitations in coding team capacity to characterize these within the time constraints of the study. The 30 narratives for the pilot study comprised text (n=15), audio (n=5) and video-based (n=10) formats.

Coders completed written evaluations of feasibility and acceptability. Findings were analyzed in the first instance by the lead author (JLB) using semantic thematic analysis,^58^ then discussed by the wider team, including researchers with a background in interaction design (SRE) and the development of psychometric measures (MS). This identified important elements missing from the inventory, and the impacts, both physical and emotional, of coding large numbers of recovery narratives. Knowledge produced by evaluation work was used to create wellbeing training and instructions for external coders. No items from INCRESE v1 were deleted, but item descriptions were refined to produce INCRESE v2.

### Stage 3: Formal Evaluation

The aim of the formal evaluation was to test (a) feasibility and acceptability to coders, (b) acceptability of INCRESE to people with lived experience of mental health difficulties and (c) the reliability of INCRESE. Twenty-two of the narratives used in Stage 2 were supplemented with 73 further narratives from the NEON Collection. The 95 narratives were a purposive sample chosen for maximum variation in format, form, length, gender, ethnicity, location, diagnosis, and collection source. This was to test whether INCRESE was applicable to the characteristics of narratives from as many different contexts as possible.

Four coders who had not previously used INCRESE and were not part of the NEON research team were recruited, comprising two from mental health research backgrounds and two from biomedical science and humanities research backgrounds, including coders with lived experience of mental health issues. The four coders were trained in the use of INCRESE v2, then each coded the same two narratives and discussed differences to assess concordance. The 95 recovery narratives were then independently coded by each coder using INCRESE v2 twice, two weeks apart (Round 1 and Round 2). Written evaluations were collected from the four coders on the feasibility and acceptability of the layout, items, responses, and descriptions in INCRESE v2. The acceptability of INCRESE v2 was reviewed by three NEON Lived Experience Advisory Panel (LEAP) members, comprising people with personal experience of mental health issues and recovery. INCRESE was finalized on the basis of statistical evaluation of poorly-performing items and further discussion of qualitative analysis findings within the wider research team.

## Analysis

### Qualitative Analysis

Written evaluations of coders and LEAP members were analyzed using a semantic approach to thematic analysis, wherein data is searched to find repeated patterns of meaning in relation to the research questions.^58^ In this study, the research questions related to the feasibility of INCRESE (e.g., aspects that were easy and difficult to use; areas for improvement) and the acceptability of INCRESE (e.g., appropriateness of language used; physical/emotional impacts on coders and people with lived experience).

### Statistical Analysis

Data were analyzed using the following criteria: (1) data completeness, (2) response distributions for each item, including floor and ceiling effects (<10% of responses), and (3) exploring the scale score distributions at each time point (Round 1 and Round 2).

To assess item-level inter-coder reliability for agreement among all four coders, Fleiss’s kappa coefficient^59^ was computed on items with nominal values (un-weighted kappa) or ordinal values (weighted kappa). Round 1 and Round 2 data were analyzed separately. Kappa value categories were defined in advance as 0.00–0.20 (None to slight); 0.21–0.40 (Fair); 0.41–0.60 (Moderate); 0.61-0.80 (Substantial); and 0.81–1.00 (Almost perfect agreement). Items with a kappa > 0.40 were identified as acceptable. To assess item-level test-retest reliability, the intra-class correlation coefficient (ICC) was calculated with a two-way random effects model. Quality of agreement was classified as: 0.00–0.19 (Poor), 0.20–0.39 (Weak), 0.40–0.59 (Moderate), 0.60–0.79 (Substantial), and 0.80-1.00 (Almost complete)^60^. Items with ICC > 0.40 were identified as acceptable. All statistical analyses were performed using SPSS (v26, IBM, Chicago, Illinois, USA).

## Results

### Stage 1: Development of Preliminary INCRESE

INCRESE v1 comprised 68 items in five sections: Narrative Eligibility, Narrative Modality, Narrator Characteristics, Narrative Characteristics, and Narrative Content.

### Stage 2: Pilot Evaluation

In relation to feasibility, coders evaluated instructions and descriptions of items as clear and thorough, with most items being straightforward to identify. Items in Section Four (Narrative Characteristics) were evaluated as more subjective and therefore more difficult to code. Some items could not be applied to all narratives, for example age of narrator in some audio-based narratives, and instructions were changed to reflect this. Other refinements to improve feasibility are shown in Table 1 (upper half).

In relation to acceptability, administrative burden was evaluated as acceptable on the whole, although some negative impacts (fatigue, loss of concentration) were reported from coding large numbers of narratives without a break. Coder wellbeing was also both positively and negatively impacted by the process of coding recovery narratives. For example, coders reported both being inspired by narratives and finding it hard to read about the painful experiences of some narrators. To address this, a section on coder wellbeing was added to INCRESE instructions, wellbeing issues were highlighted and strategies suggested in INCRESE v2 training, and debriefs were offered to coders during the formal evaluation.

The language used in INCRESE v1 was found to be more appropriate for psychometric measures than an inventory of narratives and was changed to address this: for example, ‘raters’ was replaced with ‘coders’ and ‘measure’ became ‘instrument.’ Other refinements to improve acceptability are shown in Table 1 (lower half). All refinements were implemented to produce INCRESE v2.

### Stage 3: Formal Evaluation

INCRESE v2 comprised 82 items in the same five sections. The sample narrative referred to in the introduction was coded as one of the 95 narratives. Worked examples of this and other narratives coded using INCRESE can be downloaded from http://www.researchintorecovery.com/increse.

In relation to feasibility, coders evaluated INCRESE v2 as easy to apply on the whole, due to the training provided and clear instructions. Items from Section 4 (Genre and Use of Metaphor), and some narrative formats (namely poems and images) were seen as more subjective and harder to code. Suggestions were made to amplify descriptions, for example including ‘self-help/own learning’ in the ‘education’ item of Section 5 (Narrative Content). Three sets of potentially overlapping items were reported in Section 5, between ‘death/threatened death,’ ‘injury/threatened injury,’ and ‘self-inflicted injury/self-neglect;’ between ‘formal peer support’ and ‘informal peer support;’ and between ‘hobbies/interests’ and ‘creative activities.’

In relation to acceptability, coders identified fatigue as a negative effect of the administrative burden involved but reported having enough time to complete the task (95 narratives coded over 5 days, i.e., 19 per day). Therefore, INCRESE was deemed acceptable in terms of administrative burden. Coders identified that item labels describing content warnings were stark and could in themselves be distressing to encounter. Members of the LEAP independently identified the same issue. Item names were changed in the final version to reflect this, using alternatives generated by the LEAP.

In relation to statistical analysis, completeness of data was very high, with <1% missing data for each item. No ceiling or floor effects were identified. The inter-coder reliability and test-retest reliability for Sections 1 (Narrative Eligibility), 2 (Narrative Mode), 3 (Narrator Characteristics) and 4 (Narrative Characteristics) are shown in Table 2. The inter-coder reliability and test-retest reliability for Section 5 (Narrative Content) is shown in Table 3.

Inter-coder reliability for round 1 coding ranged from 0.58 (Moderate) to 1.00 (Almost perfect), and for round 2 coding ranged from 0.63 (Substantial) to 1.00 (Almost perfect), with the exception of one outlier item #75 (Previous life circumstances) which had only Fair agreement. Coder 4 had the most variance in coding from round 1 and round 2. When controlling for coder 4, eight items showed anomalous behaviour (items #22, #28, #34, #41, #44, #45, #49 and #71). Overall, 126 (79%) of the 160 item-level ICCs were >= 0.75, indicating inter-coder reliability is strong.

Test-retest reliability (TRR) controlling for coder ranged from 0.41 (Moderate) to 1.00 (Almost complete), with the exception of the same outlier item #75 which had only Poor agreement. TRR not controlling for coder ranged from 0.57 (Moderate) to 1.00 (Almost complete), again with the exception of item #75 which had only Weak agreement. Three items performed less well in terms of TRR: #22, #34 and #38. Overall, most items had a test-retest reliability of greater than 0.75, indicating test-retest reliability is strong.

Coders had different levels of test-retest agreement, with the highest for coder 2 (mean 0.78, SD=0.25), followed by coder 3 (mean 0.71, SD=0.22), coder 1 (mean 0.64, SD=0.25) and coder 4 (mean 0.57, SD=0.27). Coder 4 was the coder with least mental health-specific experience or training.

All item names, responses, and descriptions were reviewed and refined, with a particular focus on items identified as problematic in the statistical analysis. For example, item #75 (Previous life circumstances) performed poorly in terms of both inter-coder and test-retest reliability so was deleted from the final version. For item #34 (Relationship with recovery), coding by the two coders with non-mental health research backgrounds was less reliable, so a definition of the specific meaning of recovery in mental health was added to the final version to address this.

All refinements made at items and response level to produce the final 77-item version of INCRESE are shown in Table 4. The final version of INCRESE can be downloaded from http://www.researchintorecovery.com/increse.

## Discussion

INCRESE has satisfactory acceptability, feasibility, and reliability across time and between coders. It comprises 77 items across five sections: Narrative Eligibility, Narrative Mode, Narrator Characteristics, Narrative Characteristics, and Narrative Content. 71 items characterize manifest content and six characterize latent content (Stage of recovery; Genre; Positioning; Tone; Relationship with recovery; and Trajectory). The inclusion of these six items links this study to other content analysis-based studies within mental health research which incorporate latent content in addition to the more traditional focus on manifest content alone.^33,61,62^

Using INCRESE can impact on the wellbeing of coders, with both positive and negative effects being reported. This links to previous work on researcher wellbeing when exploring distressing topics,^63–65^ on vicarious traumatization among mental health professionals and researchers,^66–68^ and on the wellbeing of online content moderators.^69,70^ Organizations utilizing INCRESE should select approaches that support coder wellbeing. Future research may focus on identifying the most effective approaches to supporting coder wellbeing when using INCRESE or similar tools, for example, by experimentally comparing different approaches to reducing harms.

### Strengths and limitations

Strengths of this study include an inclusive definition of recovery narratives, a strong theoretical basis for each included item, the use of coders from diverse backgrounds, thorough ethical consideration of both coder wellbeing and the issues raised by characterizing narratives, the coding of a large number of narratives, the inclusion of a pilot stage as well as a formal evaluation, and the testing of both inter-coder and test-retest reliability.

Limitations include that INCRESE is not suitable for use with group or collective narratives. INCRESE was not tested on longer recovery narratives such as documentaries or memoirs, so validating the inventory against such material could be a future research study. No mechanism was included in the study to assess the effects of coder fatigue; future research may explore this. Lastly, some items within the inventory are more difficult to apply to image-based recovery narratives and to more symbolic forms of prose, such as poetry. Other tools may need to be developed which can characterize such narratives more meaningfully. This is particularly important as evidence suggests that more symbolic forms may better support meaning-making for particular forms of mental health distress, as a recent conceptual review on understanding psychosis through poetry suggests.^71^

### Implications

Five uses for the Inventory of Characteristics of Recovery Stories (INCRESE) are envisaged.

First, INCRESE can be used to create catalogues of narratives for both mental health practitioners and individuals experiencing mental health distress to draw on. This would facilitate the choosing of narratives judged likely to be most helpful at a given time, with the aim of maximising benefit and minimizing harm for the recipient.

Second, INCRESE can characterize collections of narratives for use in narrative-based online interventions. Within the NEON study INCRESE will be used to characterize a large database of narratives called the NEON Collection, to enable participants to browse stories by categories. In addition, a machine learning algorithm will use the data produced by INCRESE to choose individual narratives to present to participants opting to be ‘matched’ to stories.^72^ Future research is planned to assess the effectiveness of the instrument in assisting individuals to find helpful stories.^72^

Third, as its name suggests, INCRESE can be used as a tool for organizations using recovery narratives in campaigns or on websites, and for curators of recovery narrative collections to consider the level of diversity within the narrative collection. The inventory will identify gaps, enabling the scope of the collection to be established and under-represented narrators or narrative types to be targeted for inclusion as appropriate. This may increase the likelihood of a positive connection with narratives for a wider variety of recipients.

Fourth, INCRESE can be used as a tool for researchers to assess the extent to which narratives available from organizations span a diverse range of narrative characteristics. For example, stories on health service websites (e.g., www.likemind.nhs.uk/your-stories) could be evaluated in relation to whether they include narratives from across the range of recovery narrative characteristics, including people who use a non-diagnostic explanatory framework (item #30), or whose recovery trajectory is not upwards (item #36). This would allow the extent to which concerns that “personal stories from consumer/survivors have been harnessed by mental health organizations to further their interests” are valid^73(p.86)^ to be empirically investigated.

Fifth, INCRESE can be used to provide evidence about particular characteristics that are more likely to be beneficial or to trigger distress in recipients. One way in which avoiding potential harm to recipients has been attempted in this field is the contested issue of providing content warnings, also known as trigger warnings, within collections of recovery narratives, particularly those online. Graphic description of subjects such as violence, death, or abuse are thought potentially to trigger distress similar to the original trauma in those with existing mental health conditions such as post-traumatic stress disorder. Content warnings have been widely used online and within education and professional training contexts^74,75^ to help people avoid the perceived harms which may be caused by certain narrative content. Although some empirical research is emerging,^18,19^ there is minimal empirical evidence on whether trigger warnings are helpful or not in managing distress for people who have experienced trauma. INCRESE could be used in future research to identify particularly beneficial or harmful characteristics for particular populations and to generate evidence-based guidance on the use of narratives and their potential benefits and harms.

This study also carries implications for designers of content warning systems. Reliability of content warning items in INCRESE ranged from moderate to almost perfect/almost complete agreement but were not perfectly reliable. Other content warnings systems may thus also not be perfectly reliable; hence, designers of content warning systems should assess and manage any lack of reliability, either through use of multiple coders, or by providing recipients with the information that the categorization may not be complete.

## Conclusion

INCRESE is a standardized and reliable inventory to characterize mental health recovery narratives, which is feasible for use by coders without a narrative research background, and acceptable to both coders and people with lived experience of mental health issues. It has the potential to be used not only to help individuals and practitioners to find the stories which may be most helpful to them, but also to enable curators and researchers to identify bias or lack of diversity within collections and narrative-based campaigns or interventions.

## Figures and Tables

**Table 1:** Refinements to improve feasibility and acceptability following pilot evaluation

INCRESE v1 Item	Issue identified	Refinement made in INCRESE v2
**FEASIBILITY**		
3. Is this a recovery narrative?	Coders applying differently - description contains two distinct constructs	Split into 2 items: ‘Does the narrative contain elements of adversity or struggle?’; ‘Does the narrative contain elements of success, strengths and/or survival?’
15-20. Disability categories	Rarely used - two items may be sufficient, e.g., ‘narrator identifies/does not identify disabilities’	Candidates for deletion – reviewed in Stage 3
31-32. Relationship with Recovery andStage of recovery	Found to be very similar, with ‘stage’ being more easily identifiable by coders within the narratives	Candidates for deletion – reviewed in Stage 3
40-46. Turning points	Coders applying differently	Instructions added. Items 41 & 46 renamed for greater clarity
59 & 60. Voluntary/involuntary use of mental health services	Insufficient items to characterize variety of mental health services used	Replaced with six items: ‘Relationship with mental health professional; ‘Formal peer support; ‘Informal peer support; ‘Psychological services; Involuntary uses of mental health services; ‘Hospitalization’
**ACCEPTABILITY**		
12. Ethnicity and 14. Sexuality	Available responses are insufficient to capture differences, and lead to ‘othering’ of narrators	Replaced with multiple response options
21-27. Diagnostic categories	Current items do not represent one coherent diagnostic model	DSM taxonomy chosen. ‘Obsessive-compulsive related’ item added. ‘Rejects diagnosis’ item added for narratives rejecting diagnosis.
Narrative content categories	Content frequently present but not captured by INCRESE	8 additional narrative content items added: Comparison with previous life experiences; spiritual/religious activities; experiences of stigma; creative activities; caring responsibilities; family experiences of mental health issues; diet/nutrition; and volunteering.

**Table 2:** Inter-coder and test-retest reliability for sections 1 to 4 of INCRESE v2

Item	Inter-coder reliability		Test-retest reliability (TRR)					
	Round 1	Round 2 2 weeks later	TRR by coder				TRR controlling for coder	TRR not controlling for coder
			Coder 1	Coder 2	Coder 3	Coder 4		
	*kappa*	*kappa*	*ICC*	*ICC*	*ICC*	*ICC*	*ICC*	*ICC (95% CI)*
1. Lived experience account?	0.79	1.00	0.46	0.48	1.00	0.48	0.66	0.79 (0.75-0.83)
2. Is this a narrative?	0.85	1.00	1.00	0.48	1.00	0.48	0.75	0.85 (0.82-0.88)
3. Narrator-defined adversity	0.62	0.76	**0.33**	1.00	1.00	**0.00**	0.47	0.64 (0.54-0.69)
4. Narrator-defined success	0.75	0.82	**0.35**	0.80	0.92	**0.32**	0.60	0.75 (0.62-0.79)
5. Written elements	0.99	1.00	0.97	1.00	1.00	0.98	0.97	0.99 (0.98-0.99)
6. Sound elements	0.99	1.00	1.00	1.00	0.95	1.00	0.98	0.99 (0.99-0.99)
7. Moving image elements	1.00	1.00	1.00	1.00	1.00	1.00	0.99	1.00 (0.96-0.97)
8. Static image elements	1.00	1.00	1.00	1.00	1.00	1.00	0.99	1.00 (0.98-0.99)
9. Total words (#)	n/a	n/a	n/a	n/a	n/a	n/a	n/a	n/a
10. Total length (mins)	n/a	n/a	n/a	n/a	n/a	n/a	n/a	n/a
11. Gender	0.90	0.90	0.99	0.91	0.93	0.76	0.83	0.90 (0.88-0.92)
12. Age	0.89	1.00	0.64	0.85	0.82	0.50	0.82	0.89 (0.99-1.00)
13. Ethnicity	0.95	1.00	0.85	0.95	0.77	0.90	0.91	0.95 (0.94-0.96)
14. Location of narrator	0.91	1.00	0.88	0.90	0.88	0.80	0.87	0.91 (0.89-0.93)
15. Sexuality	0.88	0.91	1.00	1.00	1.00	1.00	0.81	0.88 (0.84-0.91)
16. Visual difficulties	1.00	1.00	1.00	1.00	1.00	1.00	1.00	1.00 (1.00-1.00)
17. Hearing difficulties	1.00	1.00	1.00	1.00	1.00	1.00	1.00	1.00 (1.00-1.00)
18. Mobility difficulties	0.71	0.78	1.00	0.80	0.49	1.00	0.56	0.71 (0.64-0.76)
19. Memory difficulties	0.75	0.81	1.00	0.66	0.75	0.49	0.59	0.75 (0.69-0.79)
20. Self-care difficulties	0.76	0.81	0.66	1.00	0.49	1.00	0.63	0.76 (0.71-0.84)
21. Communication difficulties	0.86	0.86	1.00	1.00	0.80	0.39	0.76	0.86 (0.83-0.89)
22. Neuro-developmental related	0.57	0.70	0.66	1.00	1.00	1.00	0.50	0.57 (0.48-0.65)
23. Eating or food-related	0.94	1.00	1.00	1.00	1.00	1.00	0.86	0.94 (0.93-0.95)
24. Mood-related	0.87	1.00	0.72	0.91	0.83	0.48	0.77	0.87 (0.84-0.89)
25. Personality-related	0.89	0.89	1.00	0.85	0.66	0.71	0.51	0.89 (0.87-0.91)
26. Obsessive-compulsive related	0.75	0.80	0.66	1.00	0.49	1.00	0.62	0.75 (0.69-0.79)
27. Schizophrenia/psychosis	0.92	0.95	0.96	0.92	0.74	0.47	0.85	0.92 (0.90-0.93)
28. Stress-related	0.60	0.70	0.47	0.65	**0.01**	**0.00**	0.43	0.60 (0.51-0.61)
29. Substance-related	0.71	0.72	**0.37**	0.49	0.73	0.72	0.58	0.71 (0.64-0.76)
30. Rejects diagnosis	0.79	0.79	0.41	0.56	0.73	0.73	0.65	0.79 (0.74-0.82)
31. Genre	0.82	0.89	0.51	0.79	0.72	**0.34**	0.69	0.82 (0.78-0.85)
32. Positioning	0.85	0.90	0.54	0.74	0.65	0.70	0.72	0.85 (0.81-0.87)
33. Tone	0.78	0.78	0.60	0.81	0.81	0.41	0.64	0.78 (0.73-0.82)
34. Relationship with recovery	0.58	0.67	**0.38**	0.74	0.56	0.41	0.41	0.58 (0.49-0.65)
35. Stage of recovery	0.88	0.88	0.64	0.74	0.81	**0.26**	0.78	0.88 (0.85-0.90)
36. Trajectory	0.83	0.83	0.56	0.75	0.80	**0.35**	0.71	0.83 (0.79-0.86)
37. Use of metaphor	0.82	0.89	0.58	0.63	0.81	0.75	0.70	0.82 (0.78-0.85)

**Bold** = below predefined item-level threshold

**Table 3:** Inter-coder and test-retest reliability for section 5 of INCRESE v2

Item	Inter-coder reliability		Test-retest reliability (TRR)					
	Round 1	Round 2 2 weeks later	TRR by coder				TRR controlling for coder	TRR not controlling for coder
			Coder 1	Coder 2	Coder 3	Coder 4		
	*kappa*	*kappa*	*ICC*	*ICC*	*ICC*	*ICC*	*ICC*	*ICC (95% CI)*
**38. Abuse or sexual violation**	0.75	0.78	0.69	0.59	0.84	**0.30**	0.72	0.75 (0.69-0.79)
39. Death or threatened death	0.77	0.79	0.42	0.59	0.69	0.73	0.60	0.77 (0.72-0.81)
40. Self-inflicted injury	0.83	0.83	0.55	0.86	0.83	0.67	0.72	0.83 (0.79-0.86)
41. Injury or threatened injury	0.66	0.68	**0.39**	0.65	0.80	**0.36**	0.48	0.66 (0.58-0.72)
42. Injustice, prejudice, discrimination	0.85	0.89	0.83	0.81	1.00	0.42	0.73	0.85 (0.81-0.87)
43. Taking charge	0.62	0.72	**0.30**	0.50	0.57	**0.27**	0.41	0.62 (0.54-0.69)
44. Interventions/support from others	0.70	0.70	**0.25**	0.65	0.55	0.50	0.52	0.69 (0.62-0.74)
45. Self-acceptance	0.68	0.74	**0.10**	0.71	**0.24**	0.57	0.52	0.68 (0.61-0.74)
46. Spiritual	0.81	0.81	0.65	0.85	0.58	0.60	0.69	0.81 (0.76-0.84)
47. Rude awakening	0.97	0.94	1.00	1.00	0.74	1.00	0.95	0.97 (0.91-0.97)
48. Deciding to live	0.93	0.90	**0.25**	0.66	**0.14**	**0.14**	0.86	0.93 (0.91-0.93)
49. Shift in identify	0.63	0.76	**0.29**	0.88	**0.20**	0.54	0.47	0.63 (0.55-0.69)
50. Pregnancy/birth	0.99	1.00	0.77	0.77	0.66	0.66	0.98	0.98 (0.95-1.00)
51. Family	0.81	0.85	0.57	0.77	0.75	0.57	0.69	0.81 (0.77-0.84)
52. Care System	1.00	1.00	1.00	1.00	1.00	1.00	0.99	1.00 (0.99-1.00)
53. Education	0.87	0.97	0.73	0.85	0.78	0.52	0.77	0.87 (0.84-0.89)
54. Friendships	0.79	0.84	0.53	0.78	0.74	0.49	0.64	0.78 (0.73-0.82)
55. Relationships	0.77	0.73	0.52	0.75	0.81	**0.33**	0.63	0.77 (0.72-0.81)
56. Housing	0.83	0.89	0.59	0.69	0.63	0.42	0.71	0.83 (0.79-0.86)
57. Income	0.84	0.85	0.47	0.92	0.79	0.49	0.72	0.84 (0.80-0.86)
58. Work	0.78	0.78	0.67	0.71	0.67	**0.21**	0.65	0.78 (0.73-0.82)
59. Criminal justice system	0.70	0.77	0.77	0.65	0.50	1.00	0.56	0.70 (0.65-0.76)
60. Diagnosis	0.80	0.86	0.60	0.71	0.57	0.51	0.66	0.80 (0.75-0.83)
61. Medication	0.86	0.85	0.80	0.82	0.70	0.60	0.75	0.86 (0.82-0.88)
62. Relationship with MH professional	0.83	0.83	0.63	0.88	0.69	0.59	0.71	0.83 (0.79-0.86)
63. Formal peer support	0.73	0.75	**0.39**	0.64	0.63	**0.33**	0.57	0.73 (0.67-0.74)
64. Informal peer support	0.78	0.82	0.44	0.62	0.69	0.61	0.65	0.78 (0.73-0.82)
65. Involuntary use of MH services	0.84	0.84	0.71	0.79	0.85	**0.36**	0.73	0.84 (0.81-0.87)
66. Hospitalization	0.78	0.73	0.78	0.83	0.67	**0.38**	0.65	0.78 (0.73-0.82)
67. Psychological services	0.78	0.75	0.63	0.78	0.53	0.43	0.63	0.78 (0.73-0.81)
68. Alternative therapies	0.82	0.84	0.61	0.73	0.84	0.65	0.70	0.82 (0.78-0.85)
69. Being in natural environments	0.71	0.78	0.50	0.72	0.50	0.48	0.56	0.71 (0.65-0.76)
70. Animals/pets	0.77	0.76	**0.38**	0.68	0.87	**0.00**	0.67	0.77 (0.71-0.81)
71. Community activities	0.63	0.63	**0.39**	0.56	0.56	0.48	0.46	0.63 (0.55-0.70)
72. Hobbies/interests	0.74	0.73	**0.31**	0.69	0.47	0.68	0.59	0.74 (0.68-0.78)
73. Physical activities	0.71	0.78	0.44	0.91	0.48	**0.38**	0.55	0.71 (0.65-0.76)
74. Political activities	0.61	0.72	0.43	0.43	0.66	0.41	0.44	0.61 (0.53-0.68)
75. Previous life circumstances	**0.26**	**0.31**	**0.09**	0.68	**0.23**	**0.18**	**0.14**	**0.26** (0.10-0.39)
76. Spiritual/religious activities	0.89	0.94	0.81	0.73	0.70	0.62	0.80	0.89 (0.86-0.91)
77. Stigma	0.79	0.84	0.74	0.79	0.60	**0.35**	0.66	0.79 (0.75-0.83)
78. Creative activities	0.82	0.82	0.64	0.80	0.63	0.69	0.70	0.82 (0.79-0.85)
79. Caring responsibilities	0.58	0.71	**0.38**	0.65	**0.39**	**0.35**	0.41	0.58 (0.49-0.66)
80. Family experiences of MH issues	0.87	0.88	0.65	0.65	0.42	0.49	0.77	0.87 (0.84-0.89)
81. Diet/Nutrition	0.81	0.88	0.59	0.62	0.56	**0.00**	0.68	0.81 (0.77-0.84)
82. Volunteering	0.89	0.94	0.74	0.91	0.85	0.68	0.82	0.89 (0.87-0.91)

**Bold** = below predefined item-level threshold

**Table 4:** Refinements to improve feasibility, acceptability and reliability following pilot evaluation

INCRESE v2 Item	Issue identified	Refinement made in final version of INCRESE
**FEASIBILITY**		
18. Memory difficulties	Items are too similar	Merged into one item: ‘cognitive difficulties’
20. Communication difficulties		
30. Rejects diagnosis	Does not capture narratives which also use non-diagnostic terms, but do not reject diagnosis	Renamed to ‘uses a non-diagnostic framework’
34. Relationship with recovery	Current item responses do not capture narratives which contain active resistance to the recovery model	Added ‘rejects recovery’ responses to both items
35. Stage of recovery		
39. Death/threatened death	Descriptions contain potentially overlapping examples	Item names and descriptions revised into more discrete categories
40. Self-inflicted injury/self-neglect		
41. Injury/threatened injury		
63. Formal peer support	Not possible to identify whether peer support is formal or informal in some narratives	Merged into one item: ‘peer support’
64. Informal peer support		
72. Hobbies/interests	Items are too similar	Merged into one item: ‘hobbies/interests/creative activities’
78. Creative activities		
**ACCEPTABILITY**		
35. Stage of recovery	May be perceived by users as too similar to 34 - Relationship with Recovery. This item based on the Stages of Recovery (STORI) instrument, thus refers more to narrator than narrative characteristics.	Moved to Section 3 ‘Narrator Characteristics’
38-42. Content warnings	Item labels too stark - potentially distressing in themselves	Renamed
41. Self-harm	Does not clearly include eating disorder behaviours, specifically identified in previous research as potentially harmful content	Renamed to ‘Self-harm including eating disorders’
74. Political activities	Item label inaccurate, as description relates to certain political activities and not others e.g., standing for election	Item renamed to ‘Activism’ to better reflect recovery narrative content
**RELIABILITY**		
48. Deciding to live	Poor performance for TRR	Item removed; description incorporated into other turning point items.
75. Previous life circumstances	Poor performance for ICR and TRR	Item removed
